# Other-Benefiting Lying Behavior in Preschool Children and Its Relation to Theory of Mind and Empathy

**DOI:** 10.3390/bs13080634

**Published:** 2023-07-30

**Authors:** Xiaoyan Zhang, Shenqinyi Wang, Ying Wang, Qiuming Zhao, Siyuan Shang, Liyang Sai

**Affiliations:** 1 Department of Psychology, Hangzhou Normal University, Hangzhou 311121, China; 2 Shengzhou Wuai Kindergarten, Shengzhou 312400, China; 3 Zhejiang Philosophy and Social Science Laboratory for Research in Early Development and Childcare, Hangzhou Normal University, Hangzhou 311121, China; 4 Zhejiang Key Laboratory for Research in Assessment of Cognitive Impairments, Hangzhou Normal University, Hangzhou 311121, China

**Keywords:** children, other-benefiting lie, theory of mind, empathy

## Abstract

The present study examined children’s lies to help others obtain benefits (other-benefiting lying) and its relation to theory of mind (ToM) and empathy among 3–5-year-old preschool children. One hundred nine children were recruited from preschools in China. A modified hide-and-seek paradigm was used to measure children’s other-benefiting lying behavior, a ToM scale was used to measure children’s ToM abilities, and an empathy scale was used to measure children’s empathy abilities. Results showed that children tended to tell more lies to help other to get benefits as age increased, and further analyses showed that this other-benefiting lying was related to children’s ToM component of false belief understanding and their cognitive empathy performance. These findings provide evidence that cognitive factors play important roles in children’s lying to help others.

## 1. Introduction

Prosocial lies refer to those lies that are told for the purpose of meeting the needs or interests of others [1]. Prosocial lies can promote trust and play an important role in maintaining good interpersonal relationships [2]. In recent years, developmental psychologists have begun to examine children’s prosocial lies, as studying children’s prosocial lies can help us better understand how children learn social norms and solve moral conflicts [3]. In the present study, we sought to examine a type of prosocial lies told to help others obtain benefits, called other-benefiting lies, among preschool children and their relation to theory of mind (ToM) and empathy.

Prosocial lying is a complex behavior because it requires an individual to choose between two opposite rules of communication [4]. Specifically, speakers are expected to be truthful with listeners to obey the rule of maxim of quality [5]. In contrast, telling prosocial lies conforms to the basic rule of communication that requires speakers to be friendly and help [6]. Prior research has shown that children as young as 3–4 years old rate prosocial lying more positively than antisocial lying, and this rating becomes more positive as age increases [7,8]. These findings suggest that children could perceive the positive aspect of prosocial lying. Recently, researchers have begun to pay substantial attention to children’s actual prosocial lying behavior. Most of the existing research focuses on children’s polite lying behavior, which is usually adopted to avoid hurting the listener’s feelings [4]. Specifically, an undesirable gift paradigm is used to assess children’s polite lying behavior. In the paradigm, children receive a gift they do not like after completing a game with the experimenter, and then the experimenter asks the children if they like the gift. Results consistently show that children as young as 3–4 years old are able to tell polite lies to the experimenter by stating they like the gift (while telling their parents that they do not like the gift) [4,9,10,11,12,13,14]. As age increases, children tend to tell more polite lies (but also see [10]) and their lying behavior becomes more sophisticated. For example, children are more capable of telling polite lies that are credible enough to deceive the experimenters as their age increases [4].

Researchers have also examined what factors play important roles in children’s polite lying behavior. Cognitive theory of children’s lying proposes that cognitive factors are essential to the emergence and development of children’s polite lying behavior [3,15]. One of the cognitive factors that researchers intensively focus on is ToM. ToM refers to the ability to understand others’ minds and to attribute others’ behaviors to the actors’ mental states (e.g., [16]). According to cognitive theory, children’s polite lying should be related to their ToM because successful lying requires creating false beliefs in others. Several studies have supported this view. For example, Lavoie et al. [14] found that children who told polite lies in the undesirable gift paradigm had higher ToM scores, and Williams et al. [13] found that children who are able to tell a convincing polite lie (i.e., semantic leakage control) showed more advanced ToM understanding. However, other studies using similar paradigms did not find a significant relation between children’s polite lying behavior and their ToM performances. For example, Wang et al. [12] found that preschoolers’ polite lying was not significantly related to their ToM performances (also see [17]).

Although substantial knowledge of children’s polite lie-telling behavior has been acquired, it is still unclear whether children tell lies to help others gain benefits (e.g., help others gain prizes) and what factors play important roles in this kind of prosocial lies. Examining children’s lying behavior aimed at helping others obtain benefits (other-benefiting lying) has great implications. First, previous research has shown that adults are more likely to lie when lying can help others gain benefits [18]. Thus, examining children’s other-benefiting lying behavior could help us understand how children learn to balance the tension between the satisfaction of fundamental conventions of being truthful and considerations of helping another [3,17]. Second, as most existing research focuses on polite lying and its relation to cognitive factors, examining children’s other-benefiting lying behavior would improve our understanding of whether the role of cognitive factors on prosocial lying varies with motivation. To date, only two studies have examined whether preschool children tell lies to help others gain benefits. For example, Talwar et al. [19] used a helping scenario paradigm to examine whether 3–5-year-old children would lie to benefit others. In the paradigm, children played a competitive game with an experimenter (Experimenter 2) for four rounds, and Experimenter 1 rewarded the winner of each round with a sticker. The game was designed to ensure that children won every round. In the final round, Experimenter 1 left the room, and Experimenter 2 asked the child to tell Experimenter 1 that he or she had won, so Experimenter 2 could earn a sticker. The results showed that, when Experimenter 1 returned and asked who had won the game, 45% of the children chose to lie to help Experimenter 2 earn the sticker, and the tendency to lie increased with age (also see [17]).

Talwar et al. [19] also examined the relation between children’s lies to help others and their false belief understanding, but neither study found a significant relation between children’s lies to help others and their false belief understanding. However, since only two studies have examined this issue, more studies are needed to confirm this null result. In addition, several previous studies have shown that preschool-aged children’s lying is related to ToM components before false belief understanding. For example, Zhao et al. [20] found that children’s lying for self-benefiting is significantly related to early ToM components such as diverse desires. It is possible that preschool-aged children’s other-benefiting lying is also related to early ToM components. Moreover, prosocial lies may require more other-oriented motivation, and previous research has found a significant correlation between altruistic behavior and empathy [21,22,23]. The ability to feel empathy refers to the ability of individuals to interpret and predict the emotional and mental state of others based on their understanding and sharing of others’ feelings [24]. Empathy not only includes emotional empathy, i.e., the ability of individuals to generate emotional experiences similar or identical to those of the protagonist, but it also includes cognitive empathy, where individuals understand and speculate on the emotional state of the protagonist in the context [24,25]. Therefore, in addition to ToM, preschoolers’ lies to help others may be related to their ability to feel empathy. Currently, only one study has examined the relation between children’s lies to help others and empathy. Specifically, Nagar et al. [26] used the same helping scenario paradigm as Talwar and found a significant positive correlation between empathy and lying to help others in 6–12-year-old children. However, it is unclear whether this relation holds for preschool children.

In summary, this study aimed to investigate 3–5-year-old children’s lying behaviour to help others and its relation to children’s ToM and empathy. We aimed to address two research questions: (1) how preschoolers’ other-benefiting lying changed with age; (2) whether ToM and empathy abilities play important roles in children’s other-benefiting lying. To address these questions, a modified version of the hide-and-seek paradigm was used to assess children’s lying behavior in this study. In the task, children hide a prize (e.g., a sticker) in one of two cups, and then the experimenter searches for the prize. If the experimenter finds it, the prize goes to the experimenter, but if the experimenter does not find it, the child successfully helps another child win the prize. Prior to the search, the experimenter asks the child in which cup the prize is hidden, and the child can mislead the experimenter by lying. In addition, a ToM scale was used to measure different kinds of ToM components such as diverse desires, diverse beliefs, knowledge access, false belief understanding, belief–emotion, and hidden emotion [27]. This is because previous research showed that preschool children’s lying behavior is also related to the ToM components before false belief understanding [28]. Based on findings from previous research [19], we hypothesized that as age increases, children are more inclined to tell lies to help another child obtain benefits (hypothesis 1). Given the role of empathy in children’s prosocial lying [29], and the findings on the relation between empathy and prosocial lying in older children [12,26], we hypothesized that children’s other-benefiting lying would be significantly correlated with empathy (hypothesis 2). Finally, since the findings on the relation between children’s prosocial lying and their ToM are controversial, we did not make hypotheses about the relation between children’s other-benefiting lying and ToM.

## 2. Methods

### 2.1. Participants

A priori test was used to compute the required sample size by G*Power 3.1 [30] with Power (1 − *β*) set to 0.95 and *α* set to 0.05, which revealed that, to detect a significant effect in the hierarchical linear regression with a medium effect size (*f* ^2^ = 0.15), 119 children would be required in total (about 40 children in each age group). We successfully recruited 109 children aged 3-to-5 years to participate in the study from two kindergartens in Zhejiang, China. There were 41 participants in the 3-year-old group (*M*_age_ = 3.64 years, *SD* = 0.25, 20 boys), 35 participants in the 4-year-old group (*M*_age_ = 4.65 years, *SD* = 0.23, 17 boys), and 33 participants in the 5-year-old group (*M*_age_ = 5.56 years, *SD* = 0.29, 17 boys). Our study obtained permission from the children’s legal guardians, as well as verbal consent from the children themselves prior to the commencement of the study. This study was approved by the university ethics review board.

### 2.2. Procedure

Participants performed tests individually in a quiet room of their kindergarten. Children were tested on one lying task, one ToM task, and one Affective Situations Test for Empathy. The order of the tasks was counterbalanced across participants.

#### 2.2.1. Hide-and-Seek Task (Other-Benefiting Lie Task)

We used a modified hide-and-seek task to assess children’s other-benefiting lies [31]. Children were informed by the experimenter that they would participate in a game on behalf of another child who could not be present. In the task, children needed to complete practice trials to learn the rules of the hide-and-seek task and then started the test trials. In the practice trials, children were required to hide a prize in one of two cups. The cups were upside down on the table, with a “window” on the side facing the children which only allowed children to see the inside of the cup. The experimenter provided a clear explanation of the game’s rules to the children. When the experimenter guessed correctly, the experimenter was declared winner of the game and was allowed to keep the prize. When the experimenter guessed incorrectly, the children were declared winners of the game and were allowed to keep the prize. After the child had hidden, the experimenter guessed where the prize was. After the experimenter had made the choice, the experimenter asked the child whether he/she could get the prize. Only when children could answer the questions correctly, they were allowed to play the formal game.

After the warm-up, children were shown a photo of another child who was of the same age and gender as them, the experimenter told the children that the child, named Huahua (for boys) or Feifei (for girls), had the opportunity to win 10 prizes by playing a hide-and-seek game. However, he/she could not come to play, so the other children could play the game on his/her behalf. If the prize hidden by the child was found by the experimenter, Huahua could not obtain the prize; otherwise, Huahua could get the prize. Then, the child played 10 formal trials to win the prizes for Huahua. In each of the 10 trials, while the experimenter had her eyes closed, the children concealed a prize inside one of two cups. Upon the children’s declaration that they had concealed the prize, the experimenter opened her eyes and inquired, “where did you stash the prize?”; the experimenter always guessed the cup that the children had indicated. When children truthfully pointed to the cup where the prize had been hidden, it was considered truth-telling (scored 0). Conversely, if they indicated the empty cup, it was regarded as lie-telling (scored 1). The lying score varied between 0 and 10.

#### 2.2.2. ToM Scale

A Chinese version of the ToM Scale [27] was used to assess the children’s ToM understanding. The Chinese edition closely resembles the North American version, with the only difference being the substitution of character and object names with ones that resonate with Chinese children [32]. The scale includes six subtasks: Diverse Desires [33,34], Diverse Beliefs [35], Knowledge Access [36], False Belief [37], Belief–Emotion [38], and Hidden Emotion [39]. Diverse desires pertain to the acknowledgement of the fact that individuals may possess desires different from one’s own, diverse beliefs pertain to the recognition of the fact that others may hold different beliefs from one’s own, knowledge access pertains to the understanding of the fact that people may not necessarily have equal access to information, false belief pertains to the understanding of the fact that others may hold incorrect beliefs about a given situation, belief–emotion pertains to the capacity to anticipate an emotional reaction within the context of a false belief, and hidden emotion pertains to the ability to predict an emotional feeling hidden or not. Each task involved a story. The experimenter read the story to the child and asked two questions, including a warm-up or control question along with its target question, and if children answered both questions correctly, they received 1 point. As an example, for the diverse desires assessment, the researcher presented two images of food—an enticing cookie and a healthy carrot—and asked the child to indicate their preference. After the child had made their choice, the researcher introduced another child named Xiaofang, stating that Xiaofang preferred the item not selected by the participant. The researcher then inquired about which item Xiaofang would choose. Hence, the cumulative scores ranged from 0 to 6 [32].

#### 2.2.3. Affective Situations Test (Empathy Task)

The Affective Situations Test [25] is widely used to test children’s empathy in the early and middle childhood years. In the current study, it was used to assess children’s cognitive and affective empathy. There were four stories used for four affects: happiness (birthday party), sadness (a lost rabbit), fear (frightening dog), and anger (the toy snatcher). Each story was recorded in a voice that contained emotion, which was consistent with the affect reflected in the story.

Four stories with pictures were shown to the child with the recording in random order. After each story, the child was asked two questions. One was “how does the protagonist feel” (cognitive empathy) and another was “how do you feel” (affective empathy). For each question, if the child’s answer corresponded to what the story had described, he/she obtained 1 point. Thus, scores for cognitive and affective empathy both ranged from 0 to 4.

#### 2.2.4. Data Analyses Plan

First, we used descriptive analyses to assess children’s other-benefiting lying frequency for each age group, and then a one-way ANOVA was conducted to examine whether children’s tendency to tell other-benefiting lies differed across age groups (hypothesis 1). Multiple comparisons were corrected by Bonferroni correction. Second, Pearson correlations and a hierarchical linear regression were conducted to examine whether children’s ToM scores and empathy scores predicted their other-benefiting lying behavior (hypothesis 2).

## 3. Result

Preliminary analyses showed no significant correlation between gender and children’s lying frequency, and between gender and cognitive abilities (*ps* > 0.10), so all reported analyses collapsed across gender.

### 3.1. Children’s Other-Benefiting Lies

**Figure 1:**
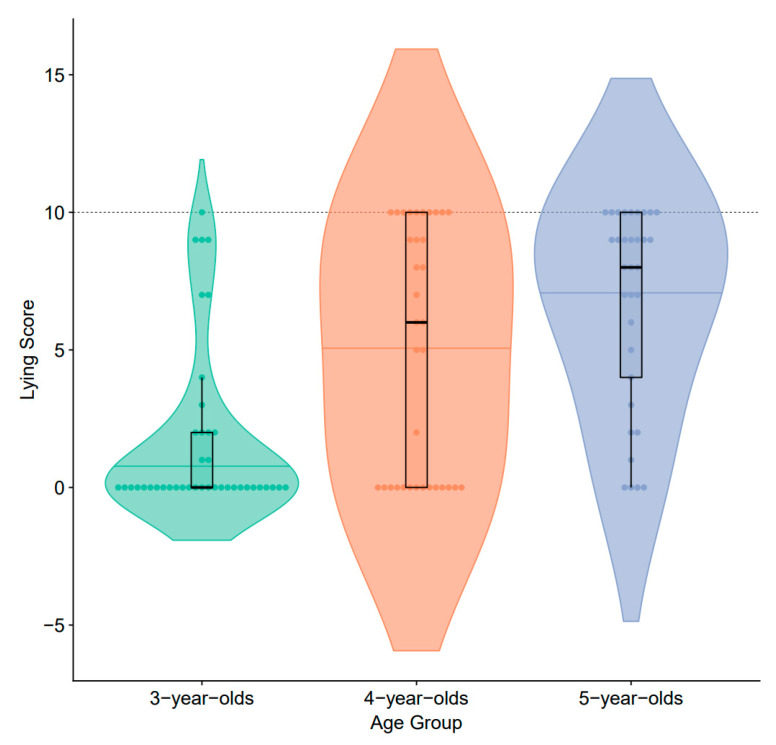
Scores of children’s other-benefiting lies in each age group.

A total of 57.7% of the children lied at least once, lying, on average, 4.25 times (10 trials in total). Specifically, the lying frequency for 3-, 4- and 5-year-olds was 1.66, 4.97, and 6.70, respectively (for details, see Figure 1).

To examine the effect of age on the children’s other-benefiting lying behavior, a one-way ANOVA was conducted with age (3, 4, and 5 years) as the independent variable and children’s lying score as the dependent variable. There was a significant main effect of the age group, *F* (2, 109) = 17.67, *p* < 0.001, $\eta_{p}^{2}$ = 0.25. Follow-up analyses with Bonferroni correction revealed that 3-year-olds told fewer lies (*M* = 1.65, *SE* = 0.48) than 4-year-olds (*M* = 4.97, *SE* = 0.76, *p* < 0.01) and 5-year-olds (*M* = 6.70, *SE* = 0.63, *p* < 0.001), while the difference between 4-year-olds and 5-year-olds’ other-benefiting lying frequency was marginally significant (*p* = 0.059) (see Table 1). This result is consistent with hypothesis 1.

### 3.2. The Relation among Children’s Other-Benefiting Lies, ToM, and Empathy

To explore the relation among children’s other-benefiting lies, ToM, and empathy, partial correlations were conducted between scores of other-benefiting lies and ToM scores, and between scores of other-benefiting lies and empathy scores. The results showed that, after controlling for age (see Table 2), children’s other-benefiting lie scores were positively correlated with their understanding of false belief (*r* = 0.27, *p* = 0.024) and their cognitive empathy scores (*r* = 0.29, *p* = 0.014).

To further examine the influence of ToM and empathy on children’s other-benefiting lies, a hierarchical linear regression was conducted with scores of other-benefiting lies as the predicted variable. Age was entered into the model in the first block, followed by scores of different ToM in the second block, and scores of empathy in the third block. Results showed that the first block was significant, Δ*F* (1, 70) = 19.82, Δ *R*^2^ = 0.23, *p* < 0.001, indicating that older children’s propensity to engage in other-benefiting lies increased (*β* = 0.48, *t* = 4.45, *p* < 0.001). The second was also significant, Δ*F* (1, 69) = 5.75, Δ*R*^2^ = 0.06, *p* = 0.019, and the remaining subscale of ToM was false belief, *β* = 0.29, *t* = 2.40, *p* = 0.019, indicating that children who had a better understanding of false belief told more other-benefiting lies. The third block was also significant, Δ*F* (1, 68) = 5.01, Δ*R*^2^ = 0.05, *p* = 0.029, and the remaining subscale of empathy was cognitive empathy, *β* = 0.25, *t* = 2.24, *p* = 0.029, indicating that children who had better cognitive empathy skills told more other-benefiting lies, which is consistent with hypothesis 2 (see Table 3).

## 4. Discussion

The present study examined other-benefiting lying behavior in 3–5-year-old preschool children and its relationship with ToM and empathy. To date, research on children’s prosocial lying behavior mostly focuses on polite lie-telling behavior. Given that lying to help others gain benefits is prevalent in adults [18], it is important to examine how this other-benefiting lying behavior develops and what factors are essential to its development. Our results showed that 3-year-old children rarely engaged in other-benefiting lies and, as age increased, 4–5-year-old children tended to engage more in other-benefiting lying behavior. In addition, we found a significant positive correlation between children’s other-benefiting lies and their ToM and empathy abilities.

First, we found that 3-year-old children told very few other-benefiting lies in the high-and-seek task (1.66 out of 10, on average), but children’s tendency to tell other-benefiting lies increased with age, and children told other-benefiting lies in more than half of the test trials at the age of 5 (6.70 out of 10, on average). These results (in line with hypothesis 1) suggest that children are more likely to tell other-benefiting lies as they grow older. This finding is consistent with research which found that children become more concerned with the needs of others as their age increases [15]. Talwar et al. [17] examined 3–6-year-old children’s other-benefiting lying and found that 45% of children were willing to tell other-benefiting lies and that children became more likely to lie as their age increased. Thus, our results pattern is similar to findings from Talwar and colleagues, although the paradigm they used is different from ours. In addition, previous studies found that children’s tendency to tell a polite lie increases with the children’s age [10]. These results together demonstrate that children’s prosocial lying increases with age regardless of whether the prosocial lying occurs to be polite or to help others gain benefits. One possible reason for this increased tendency to exhibit prosocial lying behavior is that, as age increases, children are more likely to value prosocial lying positively and behaving accordingly [8]. Further studies should investigate the relation between children’s actual other-benefiting lying and their judgment about that kind of lying to test this hypothesis.

Second, we found that children’s ToM significantly predicted their other-benefiting lying behavior. Specifically, the false belief understanding component of ToM predicted other-benefiting lying in preschool children. This is because children require the ability to implant a false belief in others to successfully deceive them [3]. This finding is consistent with previous research which showed that children’s prosocial lying such as polite lying is related to false belief understanding [8]. It should be noted that previous research has shown a significant positive correlation between preschool children’s antisocial lies and false belief understanding [40]. These results suggest that lying, regardless of the motivation, requires the cognitive process of ToM. However, our result is not consistent with the findings from Talwar et al. [19], in which they did not find a significant correlation between children’s other-benefiting lying and false belief understanding. One possible explanation is that, in their study, other-benefiting lying required children to compromise their interests, which may have led even children with ToM abilities not to engage in other-benefiting lying; hence, the lack of correlation between the two.

In addition, we found that preschool children’s empathy, especially cognitive empathy, predicted their other-benefiting lying behavior, a finding which supports hypothesis 2. Specifically, children who could understand others’ emotional states were more likely to engage in other-benefiting lying. In the current paradigm, children are aware that the child who did not come to play will be happy/sad if he/she can get/not get the stickers. Understanding these emotional states may drive children to tell lies to help the child obtain the sticker. This result is consistent with previous findings. For example, Nagar et al. [26] found that other-benefiting lying in 7–11-year-old children was related to cognitive empathy, indicating that cognitive empathy may be an internal motivation for altruistic lying [23,26]. The present findings suggest that young children are similar to school-age children in that their other-benefiting lying behavior is also related to cognitive empathy. This discovery suggests that cognitive empathy may reflect children’s other-oriented motivation to some extent [11,26].

There are some limitations to this study. Firstly, the results were based on the cultural background of China. Cross-cultural studies have found that children’s moral understanding and evaluation of prosocial lies differ between the Chinese and Western cultural backgrounds [8]. Given the significant positive correlation between lying behavior and moral evaluation of lying, future studies should further explore the influence of different cultures on children’s other-benefiting lying behavior. Secondly, this study was a cross-sectional study. Future longitudinal studies are required to explore the possible causal relationship among ToM, empathy, and other-benefiting lying in children (e.g., [17]). Thirdly, several previous studies have shown that children’s prosocial lying behavior is related to their executive function abilities. For example, Williams [13] found that 6–12-years-old children’s prosocial lying is significantly correlated to their inhibitory control and working memory abilities. Future studies should also examine the relation between executive function abilities and children’s other-benefiting lying. Lastly, this study only examined cognitive factors (ToM and empathy) that may affect children’s other-benefiting lying behavior, but previous research has shown that social and situational factors such as social class can also affect children’s lying behavior [41]. Thus, future studies should further explore how social and situational factors influence children’s other-benefiting lying.

In summary, research on children’s other-benefiting lying behavior is helpful in understanding how children resolve conflicts between different moral norms, such as honesty versus helping others. The present study found that 3-year-old children were less likely to tell other-benefiting lies, but the tendency to tell other-benefiting lies increased significantly with age, with 5-year-old children telling other-benefiting lies in more than half of the total trials. Furthermore, this study found positive correlations among children’s other-benefiting lying and their ToM understanding and cognitive empathy ability, indicating that cognitive factors play important roles in the age-related changes in children’s other-benefiting lying. These results not only provide evidence of how cognitive factors influence children’s other-benefiting lying, but also have important implications for teachers and parents in relation to the guiding of children in the development of a correct understanding of other-benefiting lies.

## Figures and Tables

**Table 1:** Lying score, ToM, and empathy by age group (*M*, *SD*).

Age Group		3-Year-Olds	4-Year-Olds	5-Year-Olds	Age Group Difference ^a^	
					*F*	*p*
	*n*	41	35	33		
Lying score		1.66 (3.05)	4.97 (4.48)	6.70 (3.63)	20.59	<0.001
ToM	Diverse Desires	0.98 (0.16)	0.91 (0.28)	0.94 (0.24)	0.30	0.742
	Diverse Beliefs	0.59 (0.50)	0.94 (0.24)	0.94 (0.24)	17.42	<0.001
	Knowledge Access	0.71 (0.46)	0.86 (0.36)	0.88 (0.33)	1.99	0.139
	False Belief	0.10 (0.30)	0.54 (0.51)	0.85 (0.36)	30.71	<0.001
	Belief Emotion	0.34 (0.48)	0.51 (0.57)	0.88 (0.33)	31.87	<0.001
	Hidden Emotion	0.17 (0.38)	0.34 (0.48)	0.30 (0.47)	1.09	0.339
Cognitive Empathy ^b^		2.33 (.82)	2.72 (1.10)	3.46 (.69)	8.96	<0.001
Affective Empathy ^b^		1.67 (.98)	2.48 (1.12)	2.96 (1.00)	7.52	0.001

Note. ^a.^ The analysis of variance (ANOVA) results for the main effect of age group. ^b.^ Some of the children could not understand the story and their data on empathy were excluded. The remaining sample sizes were 15, 29, and 28 for each age group. The main effect of the age group was tested.

**Table 2:** Partial correlations between other-benefiting lying score, ToM, and empathy (controlling for age).

	1	2	3	4	5	6	7	8	9
1. Lying score	–								
2. Diverse desires	0.08	–							
3. Diverse beliefs	0.19	0.18	–						
4. Knowledge access	0.13	−0.03	0.07	–					
5. False belief	0.27 *	0.06	0.16	0.06	–				
6. Belief emotion	0.14	0.05	0.12	−0.02	0.23	–			
7. Hidden emotion	−0.07	−0.15	−0.26 *	0.16	0.08	−0.06	–		
8. Cognitive empathy	0.29 *	0.14	−0.14	0.06	0.06	0.21	−0.05	–	
9. Affective empathy	0.14	−0.03	−0.06	−0.05	0.26 *	0.02	−0.23	0.33 **	–

Note. * *p* < 0.05, ** *p* < 0.01.

**Table 3:** The results of the hierarchical linear regression on other-benefiting lying scores.

	Variable	*β*	*t*	*p*	Δ*R*^2^	Δ*F*
Block 1	Age	0.48	4.45	<0.001	0.23	19.82 ***
Block 2	False belief	0.29	2.40	0.019	0.06	5.75 *
Block 3	Cognitive empathy	0.25	2.24	0.029	0.05	5.01 *

Note. * *p* < 0.05, *** *p* < 0.001.

## Data Availability

The data will be available on request from the corresponding author.
